# Severity and prevalence of various types of mental ill-health in a general adult population: age and sex differences

**DOI:** 10.1186/s12888-020-02557-5

**Published:** 2020-05-11

**Authors:** Per Höglund, Camilla Hakelind, Steven Nordin

**Affiliations:** Department of Psychology, Umeå University, 90187 Umeå, Sweden

**Keywords:** Burnout, Epidemiology, Insomnia, Negative affect, Somatization

## Abstract

**Background:**

Taking a broad approach, the aim of this study was to better understand severity and prevalence of various types of mental ill-health across age and sex groups in the general adult population. A first objective was to determine symptom severity of anxiety, depression, insomnia, burnout and somatization in combinations of different age groups and sex. A second objective was to determine prevalence of caseness of these types of mental ill-health in both absolute and relative terms in the combinations of age groups and sex.

**Methods:**

Cross-sectional data based on validated questionnaire instruments were used from the Västerbotten Environmental Health Study in Sweden. In total, 3406 participants, aged 18 to 79 years, constituted a random sample stratified for age and sex.

**Results:**

Severity and prevalence of anxiety, insomnia and burnout were high in women, in particular young women, and lower in older age groups. The prevalence rates for insomnia, burnout and somatization were particularly high based on the used cut-off scores. Men aged 30–49 years had the highest prevalence of mental ill-health compared to other age groups among men. Men and women aged 60–69 years had generally the lowest symptom severity and caseness. The prevalence of depression was similar in men and women in all age groups, whereas sex-related differences in extent in general were largest in the youngest age group, and gradually decreased with age.

**Conclusion:**

The results suggest that focus in primary healthcare regarding mental ill-health should to be directed more towards insomnia, burnout and somatization, in particular in young women.

## Background

Mental ill-health is an increasing public health problem. A WHO [1] report estimates that one in four persons worldwide will be affected by mental disorders at some point in life, placing these disorders among the primary causes of ill-health, with a prevalence of 23–34% [1–4]. An early study showed that Sweden was placed on average in comparison with other industrialized countries regarding this prevalence [5]. Psychiatric diagnoses such as depression, anxiety and acute stress reactions are since 2014 the most common cause of sick leave in Sweden, with a prevalence rate of about 42%. Societal and political actions may partly account for this increase in prevalence [6].

Whereas internalizing symptoms (e.g. anxiety, depression and long-term stress) are more common in women than in men [7], men are at higher risk for externalization symptoms, such as aggressive behavior and drug abuse [3, 5] as well as oppositional defiant disorder and attention deficit hyper activity disorder [8, 9]. In addition to large gender difference in prevalence rate, there are large differences between countries, which emphasizes the need of country-specific surveys. Baumeister and Härter [10] reviewed data on twelve-month prevalence rates from Australian, German, Dutch and US national surveys with comparable methodology, showing rates of 5.6–18.1% for anxiety disorders and 6.6–11.9% (4.4% for men and 7.2% for women) for major depressive disorders [10], illustrating the country-specific differences in prevalence. A Swedish general population study showed a point prevalence rate for major depression of 5.2% (3.4% for men and 6.7% for women) [11].

Insomnia and burnout are not only common in anxiety and depression [12–14], but also highly prevalent in the general population. Insomnia can be referred predominantly to unsatisfactory sleep quality or quantity and impaired daytime performance. Depending on sex, country and definition of the condition, 10–35% of the adult population suffer from disturbed sleep, and 5–10% meet the DSM-criteria for insomnia, with higher prevalence rates in women [15–19]. Burnout is an affective response to stress, and can be defined as a multidimensional construct consisting of emotional exhaustion, physical fatigue, and cognitive weariness, which together represent the core components of burnout [20]. Reported prevalence rates for burnout are 7.1–12.9% (4.6% for men and 9.3% for women) [21, 22].

Persons seeking primary healthcare for somatic concerns often exhibits anxiety, depression, insomnia and burnout [23], which calls for a broad approach that includes somatization. This condition can be defined as a tendency to experience and communicate psychological distress in the form of somatic symptoms and to seek medical help for them [24, 25], and is one of the most common problems in healthcare [10, 26, 27]. Common symptoms are fatigue, low energy, sleep difficulties, pain (in back, head, abdomen and chest), and gastrointestinal problems. An estimated prevalence of somatization syndromes in the general population is 6.3% according to a systematic review of somatoform symptoms [28]. When using the Patient Health Questionnaire 15-Item Somatic Symptom Severity Scale (PHQ-15), Kocalevent and colleagues [29] estimated the prevalence in a general population to 9.3%, with significantly higher rates for women [29]. In primary care settings the prevalence of somatoform disorder ranges from 8.6 to 25% depending on definition, design and instrument for assessment [30, 31]. One study in a healthcare setting reported a prevalence as high as 30.3% [32].

A large majority of prior studies on these issues have investigated single, or possibly pairs of aspects of mental ill-health. Thus, there is a clear lack of studies that have taken a broad approach to simultaneously assess various aspects of mental ill-health regarding both severity and prevalence across age groups and sex in the general population. This would enable direct comparisons between types of mental ill-health, age and sex groups. Given that, a first objective of the present study was to compare different types of mental ill-health in combinations of different age groups and sex with respect to symptom severity. A second objective was to determine prevalence of caseness of the different types of mental ill-health in combinations of different age groups and sex in both absolute and relative terms. In the relative comparison the different combinations of age and sex were directly compared with the group combination with the lowest prevalence. The different types of mental ill-health were anxiety, depression, insomnia, burnout and somatization. Caseness refers to scores meeting a certain cut-off, and represents high probability of being a case [33].

## Method

### Population and sample

The present study was conducted within the Västerbotten Environmental Health Study, which has a longitudinal design for the study of various forms of health issues in a general population in Sweden [34]. The present study used cross-sectional data from the first data collection in the spring of 2010. A random sample of individuals from a municipal register, aged 18–79 years, was invited to participate. The sample was stratified for sex and the following age groups: 18–29, 30–39, 40–49, 50–59, 60–69, and 70–79 years. The sample size was estimated to fully 8530 participants which was rounded up to 8600. The age and sex distribution in the sample is similar to that of Sweden in general [6, 35]. Eighty persons were excluded because they were identified as unknown by the post office, resulting in a sample size of 8520 persons.

The questionnaire was sent by mail together with written information concerning confidentiality, intended use of the data, and information about participation being voluntary. A reminder was sent to non-responders after fully 3 weeks. An additional reminder and a new copy of the questionnaire were sent after another 3 weeks. In a few cases personal at institutions for elderly assisted the elderly participant in responding to the questionnaire. Of the 8520 individuals, 3406 (40.0%) participated. The number of responders and response rates for the age groups and sex are given in Table 1. The highest non-response rate is found among men aged 18–29 years. Prevalence rates for diagnoses are given in Table 2, based on the question *Have you been diagnosed by a physician for any of the conditions below?* This was followed by 34 common somatic and psychiatric diagnoses for the participant to respond to. Perceived stress in Table 2 was measured with the Swedish version of the Perceived Stress Scale (PSS-10) [37].

**Table 1:** Number of responders and response percentages in parentheses

Age group (years)	Women	Men	All
18-29	307 (32.1)	179 (17.3)	486 (24.2)
30-39	266 (40.3)	177 (24.7)	443 (32.2)
40-49	288 (40.5)	230 (31.0)	518 (35.7)
50-59	367 (50.9)	295 (39.5)	662 (45.1)
60-69	405 (58.4)	356 (50.7)	761 (54.6)
70-79	265 (53.8)	271 (63.6)	536 (58.3)
Total	1898 (45.2)	1508 (34.9)	3406 (40.0)

**Table 2:** Description of the participants’ demographics, lifestyle and diagnoses with percentage in parentheses

	Age (years)			Sex		All
	18–39 (***n*** = 929)	40–59 (***n*** = 1180)	60–79 (***n*** = 1297)	Women (***n*** = 1898)	Men (***n*** = 1508)	(***n*** = 3406)
Married/living with partner	740 (80)***	1015 (87)	989 (77)	1522 (81)^ns^	1222 (82)	2744 (81)
Education						
Primary school	39 (4)***	136 (12)	648 (51)	425 (23)***	398 (27)	823 (24)
Senior high school	347 (38)	511 (44)	279 (22)	565 (30)	572 (38)	1137 (34)
University	533 (58)	524 (44)	348 (27)	884 (47)	521 (35)	1405 (42)
Smoking regulary	50 (5)***	122 (10)	126 (10)	186 (10)*	112 (7)	298 (9)
Physical exercise ≥ 2 times a week	558 (61)***	748 (64)	949 (74)	1345 (72)***	910 (61)	2255 (67)
Alcohol consumption ≥ 2 times a week	52 (5.6)***	189 (16)	185 (14)	212 (11)***	214 (14)	426 (12)
Perceived stress^†^	156 (28)***	147 (15)	95 (9)	273 (17)***	125 (10)	398 (14)
Excellent, very good or good self-rated health	802 (87)***	914 (78)	785 (31)	1514 (75)^ns^	1135 (75)	2501 (74)
**Self-reported physician-based diagnosis**						
Generalized anxiety disorder	9 (0.9)^ns^	14 (1)	9 (0.6)	23 (1)^ns^	9 (0.5)	32 (0.9)
Depression	65 (1.9)***	68 (2.0)	37 (1.1)	125 (3.7)***	45 (1.3)	170 (5.0)
Exhausion syndrome	22 (0.6)***	79 (2.3)	43 (1.3)	109 (3.2)***	35 (1)	144 (4.2)
Panic disorder	21 (0.6)**	21 (0.6)	8 (0.2)	36 (1.1)*	14 (0.4)	50 (1.5)
PTSD	4 (0.1)**	17 (0.5)	6 (0.2)	23 (0.7)**	4 (0.1)	27 (0.8)
ADHD	9 (0.3)*	7 (0.2)	1 (0.03)	9 (0.3) ns	8 (0.2)	17 (0.5)

Differences between age groups and sex were tested with chi-square analysis

*p < .05; **p < .01, ***p < .001, ^ns^non-significant

† Percived Stress Scale [36]

### Questionnaire instruments

The Hospital Anxiety and Depression Scale (HADS) was used, which has two subscales that measure symptoms of anxiety (HADS-A) and depression (HADS-D) during the past week based on seven items for each subscale. The items are responded to on a scale ranging from 0 to 3. A total score of 21 can be calculated for each subscale, with high score representing high level of anxiety and depression. A cut-off score of 8+ for both subscales has been shown to be the optimal balance between sensitivity and specificity for caseness (meeting cut-off for high probability of being a case) for depression or anxiety disorders [38]. The HADS has satisfactory internal consistency and concurrent validity [38, 39]. The internal consistency for the current sample was 0.85 for the HADS-A and 0.83 for the HADS-D.

Insomnia was assessed with seven items of the two dimensions sleep quality and non-restorative sleep of the Swedish version of the Karolinska Sleep Questionnaire (KSQ) [40, 41]. The KSQ has good reliability, construct validity, and criterion validity [42]. The dimension of sleep quality measures disturbed sleep, premature awakenings, and repeated awakenings with difficulty going back to sleep. The dimension of non-restorative sleep consists of questions about not being well rested on awakening and difficulties waking up. Each item in the insomnia index was dichotomized to either response alternatives 1–3 (two times or less per week) or 4–5 (three times or more per week) to reflect poor sleep, according to the DSM-5 criteria for insomnia. The index was summed and, again, dichotomized to either 0 (no symptoms) or 1 (one or more symptoms) [36]. The sleep difficulties were referred to the past 3 months, according to the DSM-5 criteria for insomnia. The internal consistency for the current sample was 0.87.

The Shirom-Melamed Burnout Questionnaire (SMBQ) was used to assess burnout, and consists of 22 items to be rated on a seven-point scale ranging from 1, “Never or almost never” to 7, “Always or almost always”. An average score across items is calculated, with high score representing high level of burnout. It measures different aspects of the burnout syndrome assessable by the dimensions of burnout, tension, listlessness, and cognitive difficulties. The SMBQ has good construct validity and reliability [43]. The internal consistency for the current sample was 0.95.

The PHQ-15 was used to assess somatization. It includes 14 of the 15 most common somatic complaints, and indicates caseness of somatization and somatoform disorder [37, 44–47]. The symptoms are rated on a scale ranging from 0 (not bothered at all), to 1 (bothered a little), and 2 (bothered a lot), giving a total score range of 0–30 for women and 0–28 for men, with high score representing high level of symptom severity. The total score is higher for women since the item “Menstrual cramps or other problems with your periods” is used only for women. For this reason, to obtain a common measure for men and women, the PHQ-15 scores were presented in percentage of total score. The items are responded to with respect to having had the symptom during the past 4 weeks. Kroenke et al. [44] recommends three cutoff points: 5, 10, and 15, representing low, medium and high somatic symptom severity. The Swedish version of the PHQ-15 which was used, which has satisfactory reliability and validity [48]. The internal consistency for the current sample was 0.82.

### Statistical analysis

Missing values were estimated with multiple imputation using fully conditional Markov chain Monte Carlo methods with 10 maximum iterations through which five imputed datasets were created, and values were averaged across datasets. The percentage of missing data were 1.3% for the HADS, 2.5% for the KSQ, 3.0% for the SMBQ, and 4.6% for the PHQ-15.

Chi-square analysis was used to test differences between age and sex groups on background variables. The alpha level was set at 0.05. Scores on the HADS-A, HADS-D, KSQ insomnia index, SMBQ and PHQ-15 were transformed to z-scores to enable direct comparison. Significant differences between age and sex groups were tested with analysis of covariance (ANCOVA) with education, living conditions (living with partner or single household), smoking, physical exercise and alcohol consumption as covariates. The alpha level was set at 0.05, and eta-square was used as effect size. Established cut-off scores with good specificity and sensitivity were used (HADS-A and HADS-D ≥ 8, SMBQ ≥4, and PHQ-15 ≥ 10) to calculate unweighted prevalence of symptoms that are clinically relevant, referred to as caseness [39, 49].

For the PHQ-15, no differences between sexes in cut-off scores has yet been recommended, despite women having a higher total score than men. Therefore, the recommended cutoff score was converted to percent of total score adjusted for men’s total score [44]. Logistic regression analyses were conducted, providing odds ratios (expressed as log_10_ for graphic illustration) for each combination of age and sex group, adjusted for education, smoking, physical exercise and alcohol consumption. The combination of age and sex group with the lowest prevalence rate was used as reference group. Thus, the odds ratios were measures of relative ratios between combinations of age and sex groups. The alpha level was set at 0.001 due to a large number of analyses.

## Results

### Sample description

The age and sex groups are described in Table 2 regarding demographics, lifestyle factors, perceived stress, self-rated health and physician-based diagnoses. Significant differences between age and/or sex groups were found for all background variables, except for the diagnosis generalized anxiety disorder.

### Symptom severity

**Fig. 1:**
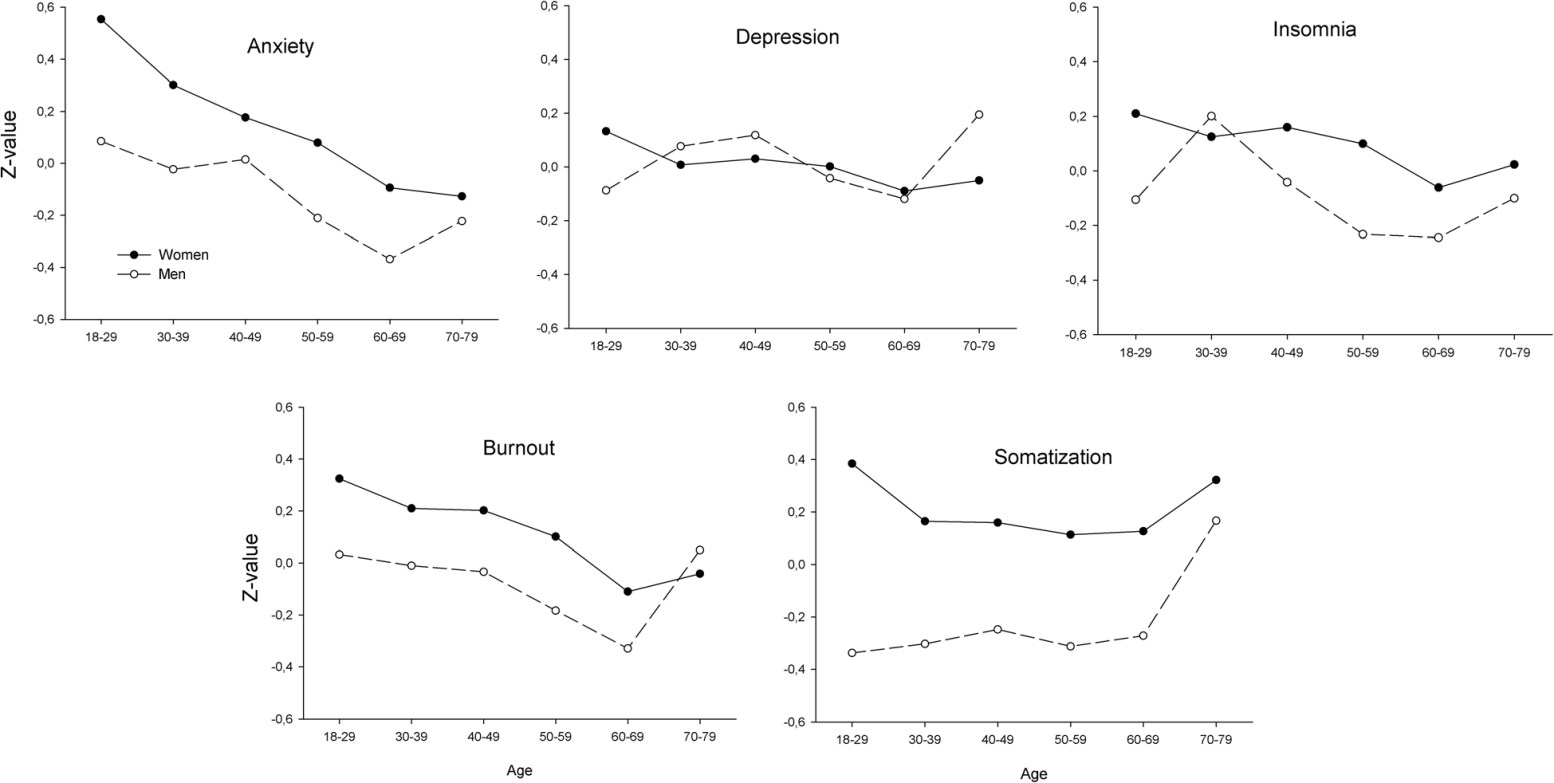
Mean z-scores for different age and sex groups on various types of mental ill-health. Standard errors range from 0.018 to 0.033

Mean z-scores across age and sex groups for anxiety, depression, insomnia, burnout and somatization are presented in Fig. 1. Standard errors are too small to be visible in the figure, and do not overlap between any combinations of age and sex group. The z-scores for anxiety and burnout among men and women were highest in the age group 18–29 years, and lower in the middle age groups, followed by higher scores in the oldest age groups. Burnout symptoms were more extensive in the age group 70–79 years, in particular in men. The z-scores for depression show high similarity between men and women, implying no clinically relevant differences in the age span 30–69 years. Symptom severity for insomnia was higher in men than in women aged 30–39 years, and was lower for both men and women in the aged 50–69 years. It was strongest in the oldest age group for men. For somatization there was a large difference between women and men in the youngest age group. Symptom severity was strongest in the age group 70–79 years for both sexes.

Results from the ANCOVAs are presented in Table 3. In accordance with the results in Fig. 1, for all five measures of mental ill-health the analyses showed significant main effects of age and sex, and age by sex interactions. However, all effect sizes were small.

**Table 3:** Results from analyses of covariance controlled for confounding variables

	Age^a^			Sex^b^			Age x sex^c^		
	*F–value*	*P–value*	*η2*	*F–value*	*P–value*	*η2*	*F–value*	*P–value*	*η2*
Anxiety	17.785	< 0.001	0.025	66.042	< 0.001	0,019	1.809	0.108	0.002
Depression	2.359	0.0380	0.003	0.306	0.580	< 0.001	2.803	0.016	0.004
Insomnia	3.620	0.003	0.005	28.901	< 0.001	0.008	1.714	0.128	0.002
Burnout	10.444	< 0.001	0.014	42.929	< 0.001	0.012	3.445	0.004	0.004
Somatization	6.210	< 0.001	0.009	116.981	< 0.001	0.033	5.290	< 0.001	0.007

^a^df = 5,3405; ^b^df = 1,3405; ^c^df = 5,17025

### Absolute prevalence of symptom caseness

**Fig. 2:**
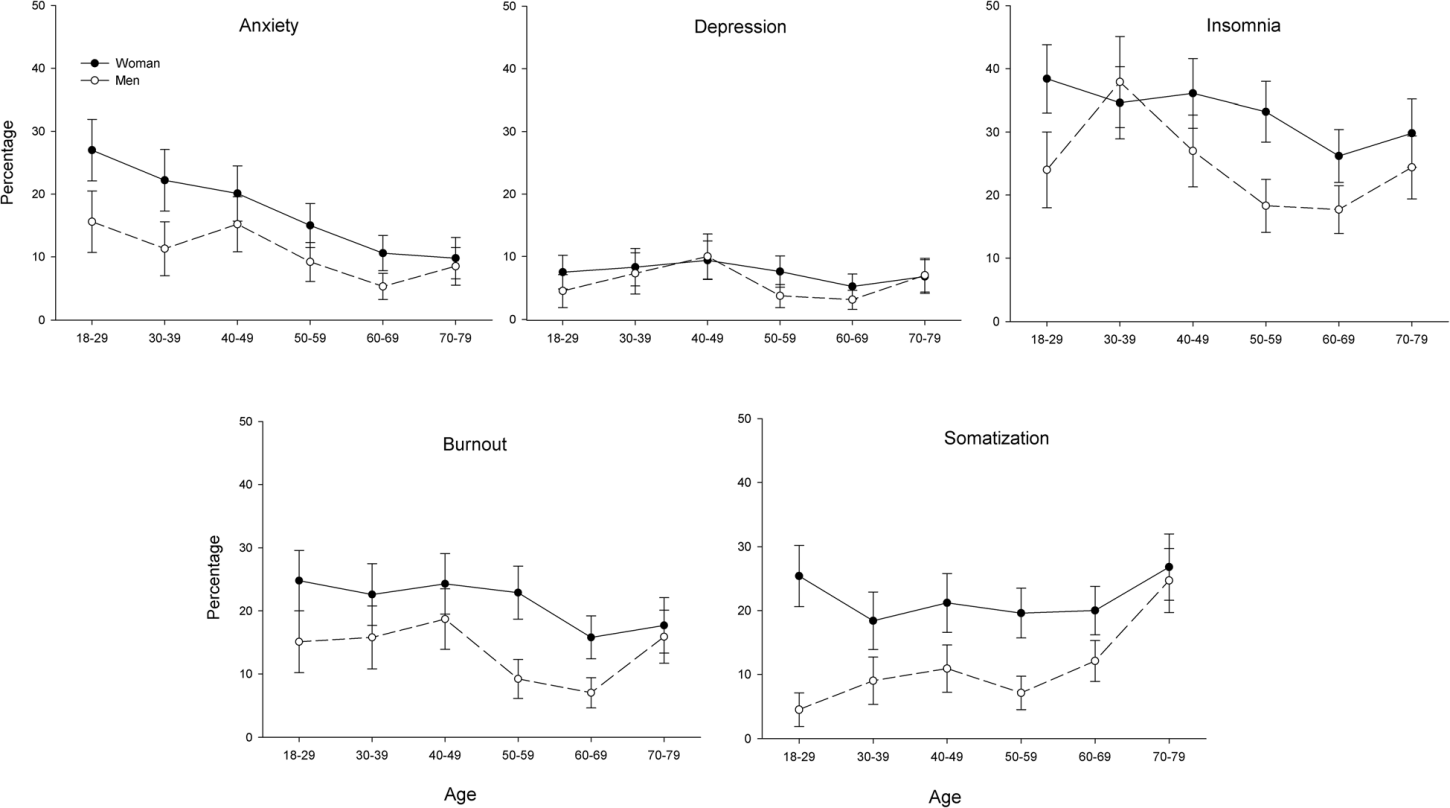
Prevalence rates (95% confidence intervals) of caseness of various types of mental ill-health for different age and sex groups

The overall prevalence rate of caseness was 13.9% for anxiety (10.0% for men, 17.0% for women), 6.4% for depression (5.5% for men, 7.1% for women), 28.6% for insomnia (23.5% for men, 32.7% for women), 17.5% for burnout (12.8% for men, 21.1 for women), and 17.4% for somatization (11.9% for men, 21.7% for women). Prevalence rates for caseness for the separate age and sex groups are presented in Fig. 2. Based on non-overlap in 95% confidence intervals, several age groups differed from each other in prevalence, especially for anxiety and somatization, as did men and women. For insomnia and burnout there was an overlap between the age group 30–39 and 40–49 years. For depression no age groups differed from each other based on the 95% confidence intervals. In the youngest age group there was a difference in prevalence between men and women in all types of mental ill-health, except for depression. In the oldest age group there was an overlap in prevalence between men and women in all types of mental ill-health.

### Relative prevalence of symptom caseness

**Fig. 3:**
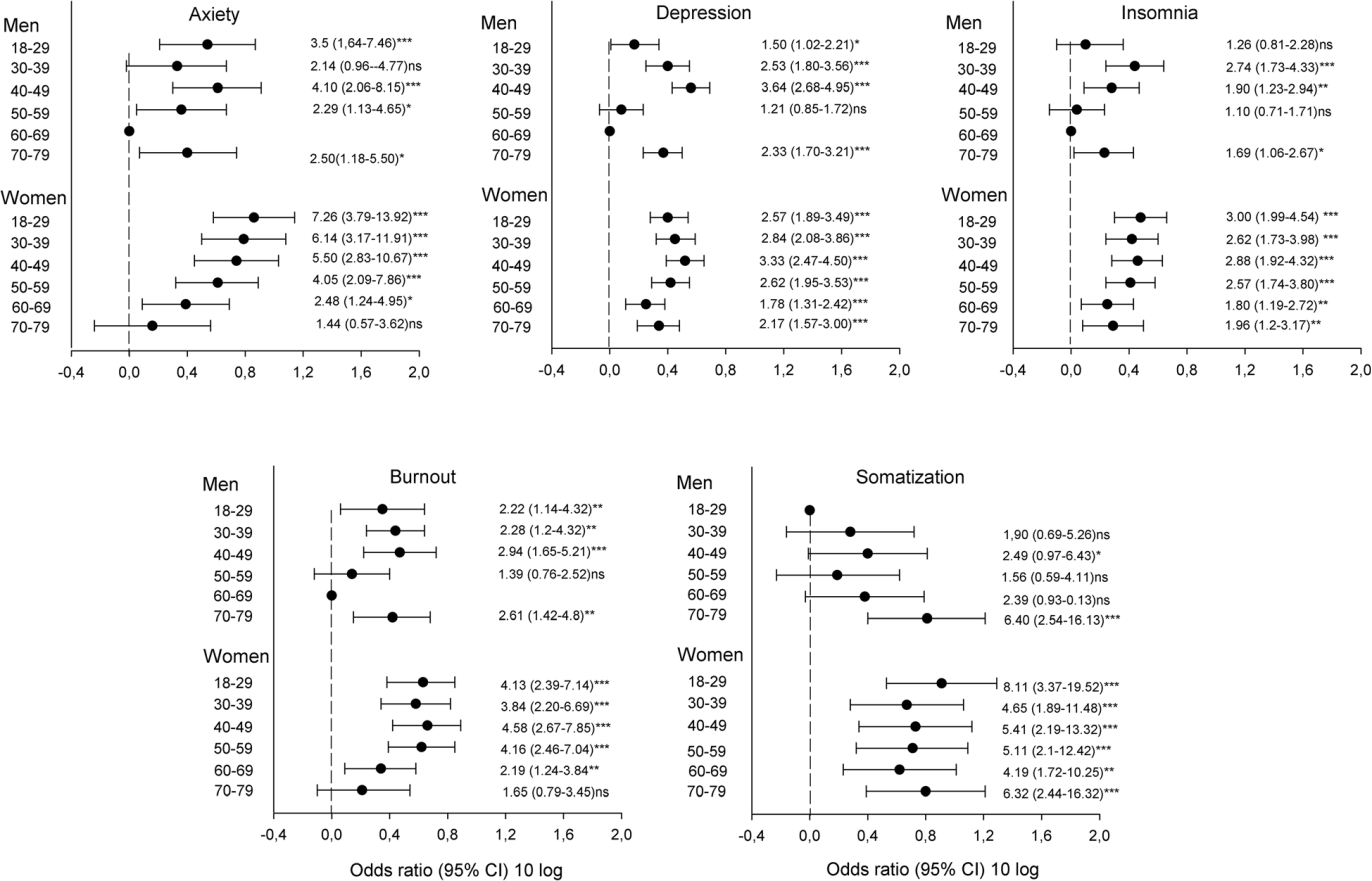
Odds ratios for caseness of mental ill-health with reference to the age and sex group with lowest prevalence, adjusted for confounding variables (*p < .05; **p < .01, ***p < .001, ^ns^non-significant)

Figure 3 displays the odds ratios for caseness of mental ill-health with reference to the age and sex group with lowest prevalence, adjusted for confounding variables. The odds ratio for anxiety was largest for women in the youngest age group. For depression the odds ratio for women and men of the same age were rather similar, except for men aged 50–69 years, who had lower odds ratio. Women were in general prone to higher risk for insomnia than men, with the highest risk for younger women, and a lower risk for older women. For men the highest risk for insomnia was in the 30–39 year age group. The risk for insomnia was lower in the age groups 50–59 and 60–69 years, and again, higher in the age group 70–79 years. The risk for burnout in women was highest in the age group 30–49 years, and lower in the age groups 60–69 and 70–79 years. The risk for somatization in women was highest in the youngest age group, and rather stable in the age group 30–69 years. In men the highest prevalence of somatization symptoms was in the age group 70–79 years. Interaction effects between age and sex were most evident in anxiety, insomnia and burnout, for which there was a general decline across age in women, whereas men were more homogeneous across age. Notably, men aged 60–69 years had lowest odds ratios for anxiety, depression, insomnia and burnout.

## Discussion

The objectives of this study were to compare different types of mental ill-health with respect to symptom severity and prevalence of caseness, the latter in both absolute and relative terms, in different age groups and in men and women. Data from this large population-based survey (*n* = 3406) showed age- and sex-related differences in mental ill-health, which in several cases were of clinical relevance. Compared to men, women in most age groups had higher levels of symptom severity and caseness prevalence. For example, there was a three-fold increased risk for somatization caseness, and a two-fold increased risk for anxiety and burnout in women in general, compared to men, which is in line with previous studies [50–54].

Interaction effects between age and sex were clearly noted regarding both symptom severity and caseness prevalence. Young women constituted a particular risk group with high levels of severity in all types of mental ill-health, and high prevalence of caseness in both absolute and relative terms, and with a fairly linear decrease with age. The prevalence of anxiety, insomnia and somatization in the youngest women was higher than that in the youngest men. This pattern has also been reported from prior studies [55–57]. In the oldest age group there were only very small differences between men and women regarding both severity and prevalence.

In men, the lowest symptom severity and caseness prevalence was, in general, found in the age group 60–69 years, whereas the oldest age group had significantly higher prevalence. It is reasonable to expect that failing physical health, social isolation, bereavement, loss of status, loss of friends and reduced income are more common among older people, thus each condition being a risk factor for mental ill-health [58, 59]. The higher prevalence of symptoms among the oldest men, regarding both depression and somatization, may be explained by overlap between certain depressive symptoms (e.g. fatigue, diminished appetite, weight loss) and common somatization symptoms [60]. Men aged 30–39 years had the highest prevalence of insomnia compared to other men, and men 40–49 years had the highest prevalence of depression and burnout caseness in both absolute and relative terms. The age group 40–49 years represents a consolidating career stage where work-life balance, family responsibilities, and ageing parents are part of a life puzzle that has to be solved [61, 62].

Shifting focus to specific types of mental ill-health, the youngest women had very high symptom severity and prevalence of caseness for anxiety, with a 7-fold increased risk for caseness compared to men aged 60–60 years. These measures of extent were lower in the oldest age group, and the differences between men and women were rather small. Findings of similar kind have been reported in the past [10, 11, 56, 63].

An unexpected result is the relatively small difference between men and women in depression. Earlier studies indicate that women experience depressive states more frequently than men, with the ratio of about 2:1 [64–66]. In this study the prevalence rate for depression was 5.5% for men and 7.1% for women, and there was no significant interaction between age and sex regarding depression, neither in severity nor prevalence. The results show a tendency of symptom severity being highest in men aged 70–79 years. However, the prevalence of depression in absolute terms shows large overlap between men and women in all age groups, making the result somewhat different to prior cross-sectional studies [67, 68]. Interestingly, similar prevalence studies in other Nordic countries using HADS show higher prevalence rate in men than in women in certain age groups [69, 70]. Whether these outcomes is a result of specific environmental factors in the Nordic countries or use of the HADS vs other instruments may be explored in future studies. It is possible that men and women simply are rather similar in this respect in the studied population. A tentative interpretation of the results in a clinical context may be that general practitioners in healthcare settings need to be more attentive to depression in men, since we know that men seek healthcare to a lesser extent, and when seeking healthcare they present to a greater extent other than psychological concerns [71].

Insomnia, was a common condition in the sample. The overall prevalence rate was 28.6% when using the sleep index [36], and was particularly high among the young and middle-aged. The prevalence was high compared to other studies, which may be due to different ways of defining poor sleep [15–19]. Men aged 30–39 years peaked in symptom severity and prevalence, with higher prevalence than in women of the same age group, but with substantial overlap for prevalence. This sex difference is in line with other studies [72–74]. Moderate sized correlation coefficients (0.40–0.53) have been reported between sleep quality and anxiety, depression, stress, and mental/physical exhaustion, which is expected since sleep quality is a common complaint in these conditions, and regarded as a transdiagnostic process in many psychiatric disorders [42, 75].

The overall prevalence of burnout (17.3%) was higher in this study than in earlier studies (7.1–12.9%) [21, 63], which partly may be explained by the relatively low cutoff score (4.0) presently used. The severity and prevalence was higher in women than in men, and decreased with age. This is coherent with mainstream theory about burnout [76]. The prevalence of burnout in men seems to have an age-span pattern that is similar to that for anxiety and depression, with a peak at 40–49 years and in the oldest age group. In contrast to Maslach’s early definition of burnout and corresponding questionnaire instruments with focus on working life, SMBQ measures burnout due to general aspects in life, and is appropriate also for older populations. Accordingly, and in line with other studies, the present data suggest a non-linear association between age and burnout [77].

The prevalence rate for somatization was higher in this study (17.4%) than in a population study by Koncalevent et al. [29] (9.3%) that also used the PHQ-15 [2, 28, 29]. The rates in this study are more in line with those from healthcare settings outside Sweden [31]. The differences across age and sex for severity was very similar to that for prevalence. Similar to other studies, women generally reported more bodily distress and more frequent somatization symptoms than men [78]. None of the age groups had an overlap in 95% confidence intervals for prevalence, except for the oldest age group, in which men had significantly higher prevalence than other age groups.

A particular strength of this study lies in its broad approach to investigate both symptom severity and caseness prevalence in both absolute and relative terms of various types of mental ill-health in different age groups and in men and women. This enables direct comparisons between the two aspects of extent, type of mental ill-health, age groups and sexes as well as their interactions in a general adult population. Other strengths of this study include being population-based, stratifying the sample for age and sex, having a large sample size, and that the study population had an age and sex distribution that is very similar to that of Sweden in general [35, 79]. The present sample (Table 2) is similar to Swedish adults in general according to national data: smoking 13%, risk alcohol consumption 17%, perceived stress 13%, regular exercise 65% and good self-rated health 73% [79].

There are also limitations to be considered. The data was collected in 2010, which limits the generalizability of mental ill-health to today. Only 40% of the sample responded to the questionnaire, compromising the representativeness. This refers, in particular, to young men, making the interpretation of results from this cohort uncertain [36]. It does also have consequences for the overall mean severity scores and prevalence rates, which are likely to be somewhat overestimated,. The sample size was large, resulting in statistically significant group differences even for small absolute differences, which highlights the importance of the effect sizes. In this study eta-square values were small, implying that age and sex do not show substantial differences in mental ill-health in a general population. This sums up that even though age and sex to some extent can explain differences in mental ill-health, complementary studies of other mediating and moderating factors are needed. Furthermore, register data on diagnoses may have provided more reliable results than presently used questionnaire instruments for assessing caseness. However, as reviewed, the reliability and validity of the instruments used are in general good.

Cross-sectional studies of this kind cannot answer the question as to whether an age-related variation in health status is due to environmental and societal change or transition phases (e.g. developmental changes) [80]. For this reason the findings may not necessarily mean that ageing itself brings about a diminution in symptoms. However, the WHO argues for higher prevalence rates for anxiety and mood disorders in more recent cohorts in many countries (Andrade et al., 2000). Additional longitudinal studies covering the adult life span are needed to differentiate ageing from cohort effects [55].

A clinical implication of the present results is that there is a gray zone of individuals outside the healthcare system who do not seek help. In this study the ratio in prevalence between those with caseness of a certain type of mental ill-health and a physician-based diagnosis (Table 2) varied from 4.17 to 6.85, indicating that these conditions are undertreated in the general Swedish population. The prevalence rates obtained in the current study are in line with prior prevalence studies in Sweden [11, 81].

Many patients with mental ill-health present to their general practitioner with common somatic symptoms that may result in their mental ill-health being missed in the assessment [71, 82, 83]. The high prevalence rates found in the current study calls for further efforts regarding development and validation of good prevention interventions and treatments in primary healthcare settings [84]. Such procedures may be particularly valuable for men since they seek healthcare for mental ill-health to a lesser extent than women [85], and are more likely to commit suicide [86–88].

## Conclusions

Some of the findings from this study are of considerable interest. The results suggest that anxiety, insomnia and burnout are particularly severe and prevalent in young women. Middle-aged men have higher prevalence of mental ill-health compared to other age groups of men, with the lowest severity and prevalence in the age span 60–69 years. Since the results show that mental ill-health is relatively common in early adulthood, early assessment and interventions in this cohort may help prevent persistence and recess of mental ill-health. Longitudinal studies covering the adult life span are needed to understand mediation and moderating factors underlying age- and sex-related risks factors for mental ill-health.

## Data Availability

Data at group level are available from the corresponding author at reasonable request.

## Abbreviations

WHO: World Health Organization
DSM: Diagnostic and Statistical Manual of Mental Disorders
PHQ-15: Patient Health Questionnaire 15-Item Somatic Symptom Severity Scale
HADS: The Hospital Anxiety and Depression Scale
KSQ: The Karolinska Sleep Questionnaire
SMBQ: The Shirom–Melamed Burnout Questionnaire
ANCOVA: Analysis of covariance
